# NEIGHBORHOOD COHESION MODERATES THE ASSOCIATION BETWEEN DEMENTIA STATUS AND SUBJECTIVE WELL-BEING

**DOI:** 10.1093/geroni/igad104.2655

**Published:** 2023-12-21

**Authors:** Dokyung Yoon, Eileen Crimmins, Jennifer Ailshire

**Affiliations:** University of Southern California, Leonard Davis School of Gerontology, Los Angeles, California, United States; University of Southern California, Los Angeles, California, United States; University of Southern California, Los Angeles, California, United States

## Abstract

Neighborhood social cohesion may act as a protective factor against the negative effects of certain stressors associated with dementia status on subjective well-being. The current study aimed to investigate the moderating effect of neighborhood social cohesion on the relationship between dementia status and subjective well-being among older adults. The status of dementia was classified as either probable or possible, or as not having dementia. Data were drawn from the 1st round (2011) of the National Health and Aging Trends Study (NHATS), a panel study of nationally representative Medicare beneficiaries aged 65 and older. The sample consisted of 5,407 community-dwelling older adults. A linear regression model indicated that dementia status was associated with a lower level of subjective well-being (b = -2.34, p < .001), while neighborhood social cohesion was associated with a higher level of subjective well-being (b = 1.04, p < .001). Furthermore, neighborhood social cohesion moderated the association between dementia status and subjective well-being (b = .67, p = .003). In older adults with probable or possible dementia, the relationship between neighborhood social cohesion and subjective well-being was observed to be stronger, as evidenced by a steeper slope. The findings of this study contribute to the growing body of evidence that emphasizes the crucial role of ties within one’s neighborhood in influencing subjective well-being. Building strong communities of individuals who rely on and trust one another may help alleviate the negative impact of probable or possible dementia on subjective well-being.

